# Neural sources of letter and Vernier acuity

**DOI:** 10.1038/s41598-020-72370-3

**Published:** 2020-09-22

**Authors:** Elham Barzegaran, Anthony M. Norcia

**Affiliations:** Wu Tsai Neurosciences Institute, 290 Jane Stanford Way, Stanford, CA 94305 USA

**Keywords:** Extrastriate cortex, Striate cortex

## Abstract

Visual acuity can be measured in many different ways, including with letters and Vernier offsets. Prior psychophysical work has suggested that the two acuities are strongly linked given that they both depend strongly on retinal eccentricity and both are similarly affected in amblyopia. Here we used high-density EEG recordings to ask whether the underlying neural sources are common as suggested by the psychophysics or distinct. To measure visual acuity for letters, we recorded evoked potentials to 3 Hz alternations between intact and scrambled text comprised of letters of varying size. To measure visual acuity for Vernier offsets, we recorded evoked potentials to 3 Hz alternations between bar gratings with and without a set of Vernier offsets. Both alternation types elicited robust activity at the 3 Hz stimulus frequency that scaled in amplitude with both letter and offset size, starting near threshold. Letter and Vernier offset responses differed in both their scalp topography and temporal dynamics. The earliest evoked responses to letters occurred on lateral occipital visual areas, predominantly over the left hemisphere. Later responses were measured at electrodes over early visual cortex, suggesting that letter structure is first extracted in second-tier extra-striate areas and that responses over early visual areas are due to feedback. Responses to Vernier offsets, by contrast, occurred first at medial occipital electrodes, with responses at later time-points being more broadly distributed—consistent with feedforward pathway mediation. The previously observed commonalities between letter and Vernier acuity may be due to common bottlenecks in early visual cortex but not because the two tasks are subserved by a common network of visual areas.

## Introduction

Over many years of clinical practice beginning in the nineteenth century, visual acuity measured with high contrast letter charts has been the primary method of assessing visual function in patients^1–4^. The task involves the identification of letters of decreasing size until a limit is reached. Visual acuity measured with letter charts is sensitive to optical defocus, media clarity and a host of neural factors making it an efficient, albeit non-specific method for detecting visual abnormalities. Starting in the middle of the twentieth century, alternative acuity targets such as gratings^5–8^ and Vernier offsets^9^ have been used to measure visual acuity, especially in the area of amblyopia research^10–12^. These alternative measures of visual acuity reduce some of the cognitive demands of letter identification and have thus been adapted for use in pre-verbal participants^13–17^.

Early on it was realized that grating acuity systematically underestimates the letter acuity loss in the case of amblyopia^11^, especially in patients who have deficient stereopsis^18^. However, a number of studies in amblyopia^11,18–20^ noted that Vernier acuity losses scale in a similar fashion as letter acuity. These findings suggested that Vernier acuity might be a useful predictor/correlate of letter acuity. Vernier acuity involves the discrimination of the relative position of nearby visual features such as oriented lines^9,21^. Coding of the relative position of features is also important for letter acuity, but Vernier acuity exceeds the fundamental sampling limit set by photoreceptor spacing^22^, while letter acuity does not. One factor that may determine the degree to which the amblyopic visual loss manifests is the eccentricity dependence of the acuity task. Grating acuity falls off relatively slowly in the periphery and its rate of fall-off is consistent with sampling limits on resolution set by the retina^23^. The much steeper dependence on eccentricity shown in both Vernier and certain forms of letter acuity^24–27^ suggests that additional constraints are imposed in visual cortex, which is also the primary site of amblyopia^28–31^.

The cortical basis of Vernier acuity has been little studied. Neurons sensitive to the presence of spatially shifted bar/grating segments in an otherwise homogenous stimulus array have been found in macaque V1^32^ and V2^33^. Recent fMRI-informed source imaging studies^34–38^ have measured Steady State Visual Evoked Potentials (SSVEPs) to similar stimuli. These studies used frequency-domain analysis^14^ to isolate Vernier-related activity and found widespread Vernier-related activity throughout early visual cortex and mid-level extra-striate visual areas, consistent with the previously documented sensitivity to Vernier offsets in early visual areas of the macaque.

While the underlying neural mechanisms and cortical areas responsible for letter acuity per se have not been determined, a large literature has studied cortical responses to highly supra-threshold letter and word forms. Some of this work has compared functional Magnetic Resonance Imaging (fMRI) activations to letter or word forms with those of non-word patterns intended to serve as controls for low-level feature responses^39–43^. Differential activations to these contrasts are found on the ventral surface of visual cortex anterior to retinotopic areas, specifically in a region referred to as the Visual Word Form Area (VWFA) and also in Inferior occipital gyrus (IOG)^42,44^. Intra-cranial recordings have also found a similar distribution of letter/word related activity on the ventral surface^44,45^.

The existing literature thus suggests that Vernier offsets are represented in early visual areas that are retinotopic with small receptive fields and that letters are represented well outside of early visual cortex, but the degree to which the two tasks may share cortical processing resources at their respective resolution limits, as suggested by their correlation in disease, has not been determined. Here we make such a comparison within subjects using a combination of data-driven source separation methods and a common SSVEP paradigm sensitive to the spatial structure inherent in both Vernier offset and letter tasks. We find that the topographies of Vernier and letter-related activity have both distinct and common aspects: the dominant response component for letter-related activity peaks on electrodes over left lateral/ventral cortex while that for Vernier offsets is maximal over the occipital pole, but the two paradigms share a second, posteriorly maximal component. In the case of letters, the lateral component leads the posterior component, suggesting feedback from second-tier extra-striate areas to early visual cortex. In the case of Vernier offsets, by contrast, the response pattern is consistent with a feedforward pathway.

## Results

**Figure 1:**
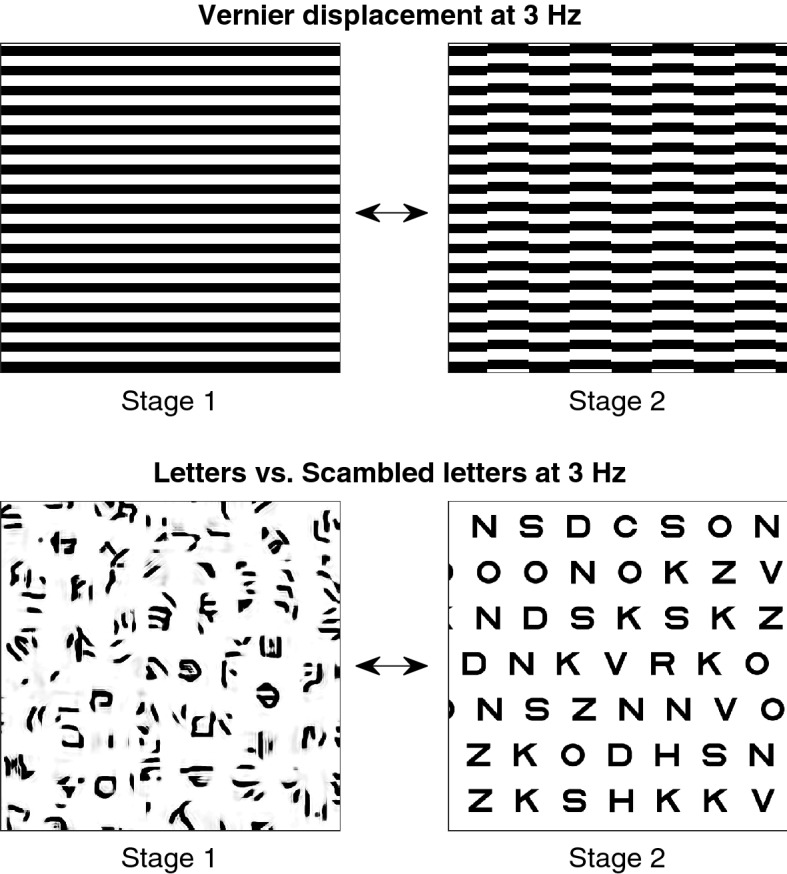
Vernier and letter stimuli. Top. Schematic illustration of the Vernier offset stimulus alternation between a base image comprising a 2 c/deg square wave grating and an offset grating of a fixed displacement. Bottom. Schematic illustration of the letter task stimulus alternation. Scrambled letter images were alternated with intact letter images of the same size. Images alternated at 3 Hz in both Vernier and letter conditions.

The letter stimulation paradigm consisted of arrays of intact and scrambled letters, alternating periodically at the rate of 3 Hz^46^. Letter arrays were used to increase the Signal-to-Noise Ratio (SNR) of the SSVEP over that possible with single letters. Secondly, an array format allowed us to employ a scrambling procedure designed to work on textures with consistent statistics over the image^47^. The scrambling procedure controls several low-level image features between intact and scrambled textures. Finally, the array format also induces spatial interference or “crowding” effects that are present in typical letter acuity charts that are used clinically. Crowding is an important factor in acuity measurement in that crowding effects are known to more strongly degrade letter acuity in young children^48^ and patients with amblyopia^49–51^*.* The Vernier stimuli consisted of a similarly large base pattern of a bar grating into which Vernier offsets were periodically introduced and withdrawn at the rate of 3 Hz^14^. The periodic grating format for the Vernier target increases SNR over that possible with single Vernier targets and may also induce crowding effects^23,50^. Schematic illustration of the stimuli is shown in Fig.1. See Methods for additional details on the visual stimuli.

### Scalp topography of letter and Vernier acuity responses

We applied harmonic analysis to the SSVEP data to separate configural activity specific to differences between intact and scrambled letters, and aligned and misaligned lines from non-configural components of evoked responses. SSVEP amplitude versus Vernier offset and letter size was measured for 5 sizes starting near their respective acuity limits.

**Figure 2:**
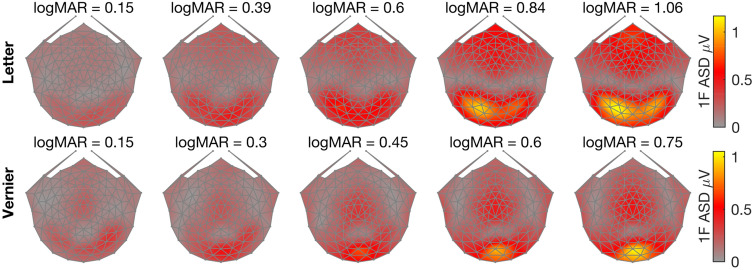
Scalp topography of 1F response amplitude for letter and Vernier targets. The stimuli in both conditions were near threshold in the leftmost panels and were well above threshold in the rightmost panel. The color bar on the right side of each row corresponds to Amplitude Spectrum Density (ASD in microvolts).

**Figure 3:**
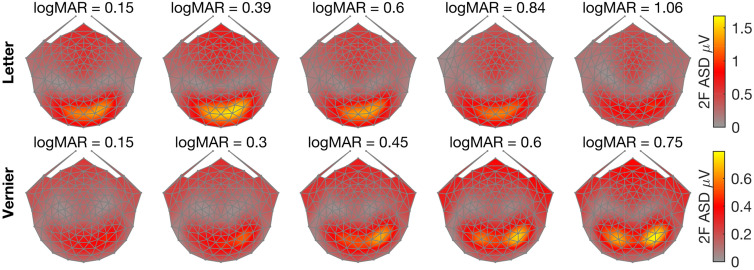
Scalp topography of 2F response amplitude for letter and Vernier targets. The stimuli in both conditions were near threshold in the leftmost panels and were well above threshold in the rightmost panel. The color bar on the right side of each row corresponds to Amplitude Spectrum Density (ASD in microvolts).

The configural responses are represented in the odd harmonics and non-configural responses in the even harmonics^52^. For illustration purposes, Figs. 2 and 3 plot the scalp topography of the first (1F) and second (2F) harmonic responses respectively as a function of the spatial scale of the letters and Vernier offsets used. Starting with the 1F response, amplitudes for both letter and Vernier stimuli increase monotonically as the size of the letters and Vernier offsets increases from near threshold values (LogMar of 0.15) to the maximal values presented (LogMar 1.06 and 0.75, respectively). Importantly, the topography of the two responses differs. Responses at 1F for letters are maximal at left lateral occipital electrodes (EGI HSN128 electrodes 65 and 66), while those to Vernier offsets are maximal and symmetrically distributed over the occipital pole (EGI HSN128 electrode 75).

The second harmonic (2F) response to letters, by contrast, is a non-monotonic function of letter size and its response topography covers medial occipital electrodes with a biased extension to right lateral electrodes (EGI HSN128 electrodes 75 and 83). The 2F responses for the Vernier target are monotonically increasing with offset size and have distinct bilateral maximum amplitudes at lateral occipital electrodes, with a right hemisphere bias (EGI HSN128 electrodes 90 and 91). Distinct topographies for letter and Vernier target are thus present for both harmonics and both stimulus types.

### Reliable component analysis (RCA)

The topography of the scalp-recorded SSVEP consists of the summation of activity from one or more underlying neural generators. The multiple distinct topographies seen in Figs. 2 and 3 indicate that more than one source underlies the 1F and 2F response topographies and that these differ for letters and Vernier offsets. To further separate underlying sources, we used the data-driven RCA approach that separates sources based on the existence of distinct patterns of trial-to-trial reliability. We concentrate on the 1F responses because they can be more readily interpreted in terms of configural, rather than local transient processing mechanisms^52^. A parallel RC analysis for the 2F responses is presented in the Supplementary Materials.

#### RCA of 1F responses

**Figure 4:**
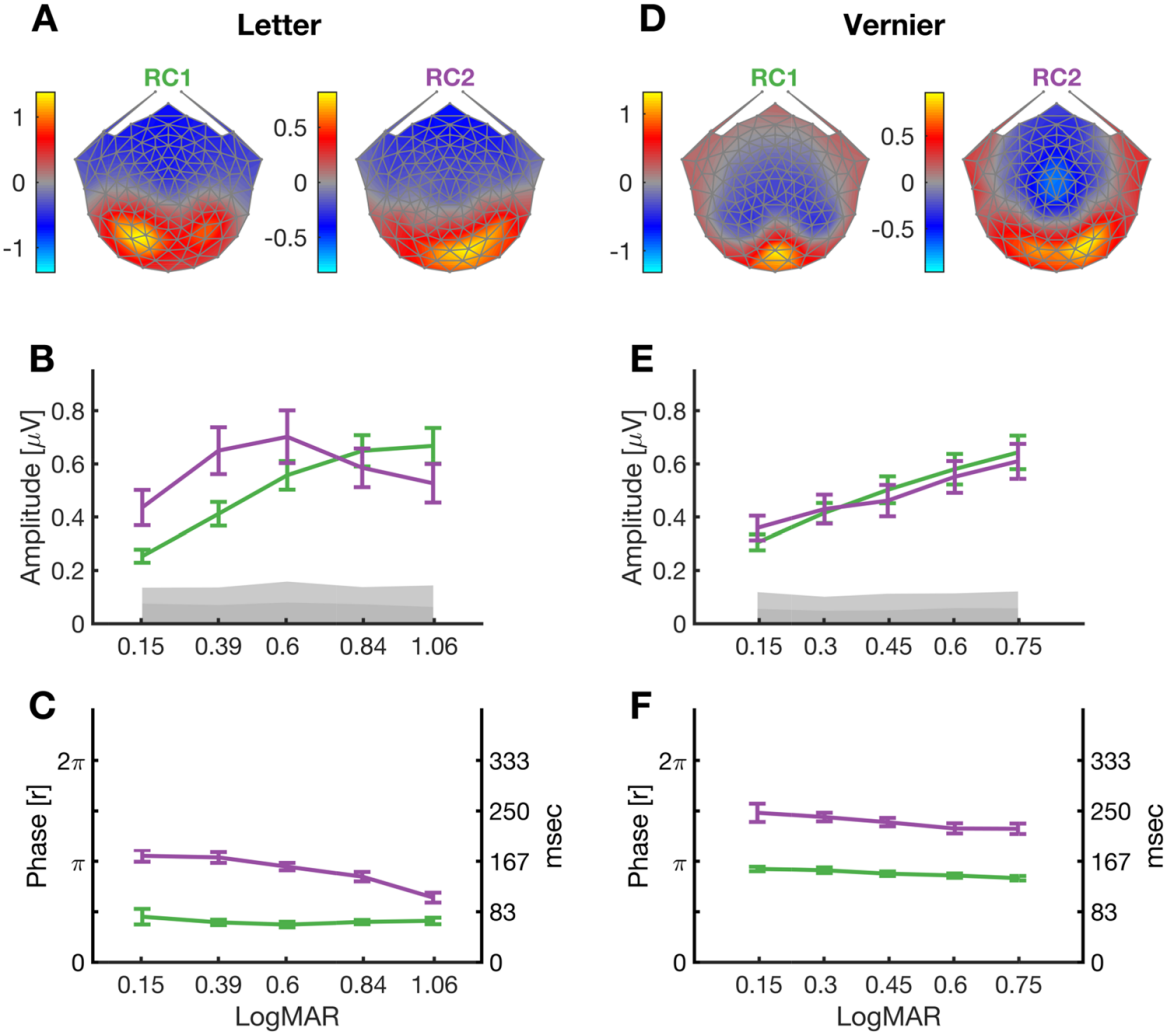
RCA weights, amplitudes and phases of 1F response component for letter and Vernier targets. (**A**) Letter task 1F RCA topography (left) is maximal over left lateral occipital electrodes, RC2 topography (right) is more posteriorly distributed over the occipital pole, with a right hemisphere bias. (**B**) Letter task 1F amplitude as a function of increasing font size for RC1 (green) and RC2 (purple). (**C**) Letter task 1F phase as a function of increasing font size for RC1 (green) and RC2 (purple). (**D**) Vernier task 1F RCA topography (left) is focally distributed over the occipital pole with a maximum at the occipital midline. RC2 topography (right) is more broadly distributed over the occipital pole, with a right hemisphere bias. (**E**) Vernier task 1F amplitude as a function of increasing offset size for RC1 (green) and RC2 (purple). (**F)** Vernier task 1F phase as a function of increasing offset size for RC1 (green) and RC2 (purple). The color bars in (**A**,**D**) indicate the weightings of RC1 and RC2 topographies. The mean noise level measured as the mean amplitude of adjacent frequency bins to 1F, e.g. the mean amplitude of 2.5 and 3.5 Hz, passed through RC1 and RC2 filters, is indicated by the grey area.

Figure 4 shows the RCA filters learned separately for the letter and Vernier 1F response components over the five letter and Vernier offset sizes. We selected the first two reliable components for further analysis as they explained around 98% of the trial-to-trial reliability in the letter and Vernier conditions. In the Letter task, RC1 (the first reliable component, shown in the left panel of Fig. 4A) has average SNR of 9.5 dB over the 5 letter sizes (*p* < 0.001) and a scalp topography that is maximal over left-lateral occipital electrodes (65 and 66), as does the raw scalp topography (Fig. 2 top panels). A second RCA component (RC2, Fig. 4A, right panel) was present with average SNR of 9.3 dB (*p* < 0.001) and is more medially distributed, with a bias to the right hemisphere (electrodes 83 and 75).

RCA partitions the total scalp topography, shown in Fig. 2, into two separate sources of activity that have different functional tuning and response phases. Figure 4B plots 1F amplitude as a function of letter size for both RC1 (green) and RC2 (purple). Both functions start with an increase in response amplitude with increasing letter size. The RC2 function has a maximum amplitude at 0.6 LogMAR, declining for larger letters. The RC1 function continues to increase in amplitude, reaching a saturated maximum at LogMAR of 0.84 and 1.06. To compare the shape of these tuning functions, we applied a two-way repeated measures ANOVA, as described in the Methods section, with amplitude as dependent variable and RC and logMAR as independent variables. The ANOVA indicated a significant interaction between RCs and the logMARs (*p* = 0.002, F_1,176_ = 9.74). The fact that the functions have different shapes independently verifies that RC1 and RC2 arise from different underlying sources as a single source would have a common response function.

The corresponding data for the Vernier RC1 and RC2 components at 1F are shown in Fig. 4D-F. Like the raw scalp topography of Fig. 2 bottom, RC1 is focally distributed over the occipital pole (electrode 75; Fig. 4D, left). The average SNR of RC1 was 9.5 dB (*p* < 0.001). The Vernier RC2 component (SNR = 8.6 dB, *p* < 0.001) is posteriorly distributed and biased to right-lateral electrodes (electrode 90). Both RC1 and RC2 response *vs* displacement functions at 1F are linearly increasing functions of log displacement. The two-way ANOVA on amplitude (with RC and logMAR as factors) showed only a main effect of logMAR (*p* < 0.001, F_1,176_ = 26.06), indicating that the RC1 and RC2 amplitude tunings were not significantly different.

#### Dynamics of the RC components at 1F

Beside the distinct topographies and response functions for RC1 and RC2, additional evidence for them arising from different sources comes from the distinct phase behavior of these components. For letter targets, the phase of RC1 at 1F is nearly constant as function of letter size, but the phase of the RC2 function shifts progressively in the direction of the phase origin (0 deg, which corresponds to stimulus onset; Fig. 4C). This means that the RC2 generator latency decreases as the letter size increases, while RC1 generator does not. This difference in phase behavior was confirmed by an ANOVA on the phase of RC1 and RC2 that showed a significant interaction between RC components and logMAR values (*p* < 0.001, F_1,176_ = 21.17).

**Figure 5:**
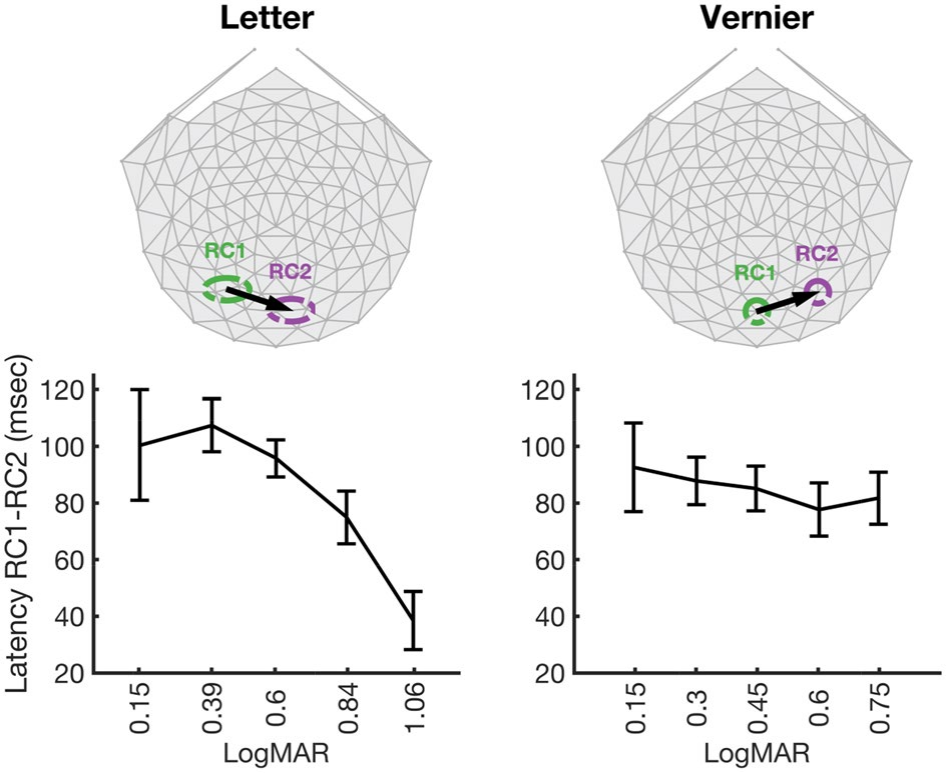
Response latency differences of RC1 and RC2 of 1F harmonic. Left. In the letter target condition, RC1 has maximum amplitudes located over electrodes 65 and 66 (green dashed ellipse) and leads RC2 which has maximum amplitudes located over electrodes 83 and 75 (purple dashed ellipse). The latency difference between RC1 and RC2 components, estimated in msec over different logMARs is presented at the bottom. Right. In the Vernier target condition, RC1 has maximum amplitude located over electrode 75 (green dashed circle) and leads RC2 which has maximum amplitude over electrode 90 (purple dashed circle). The latency differences between RC1 and RC2 are presented as a function of Vernier offset size, at the bottom.

To more directly compare the relative phases of RC1 and RC2, and to derive their order of processing through cortex, we computed the difference in phase at each letter size, as shown in Fig. 5. For letters, RC1, which peaks at left lateral electrodes (electrodes 65 and 66, green dashed circle) leads RC2, which peaks at electrodes 83 and 75 (purple circle). This relative delay is ~ 100 ms at small letter sizes, decreasing to ~ 40 ms as letter size increases. This pattern of phase delays is consistent with the response sensitive to letter structure, as indexed by 1F, being generated first in extra-striate areas and then appearing via feedback at a later time on electrodes over early visual areas.

For the Vernier targets, the phase of RC1 and RC2 at 1F shows a small phase lead going from small to large offsets, with a constant phase difference between them at all offset sizes (Fig. 4F). The two-way ANOVA on phase (with RC and logMAR as factors) showed a significant main effect of RC (*p* < 0.001, F_1,176_ = 78.26), indicating that the phase of RC1 and RC2 were on average different, but their tunings as a function of logMAR values were not significantly different.

We also computed the phase difference at each Vernier offset. There is a constant ~ 90 ms phase lag between RC1 which peaks on electrode over early visual areas and RC2 which peaks at electrode over right lateral visual areas (Fig. 5). This pattern is consistent with the response sensitive to Vernier structure, as indexed by 1F, being generated first in early visual areas and being fed forward to extra-striate areas, especially to those in the right hemisphere. Component analysis for the 2F component showed activation patterns consistent with feedforward processing for both RC1 and RC2 (see supplementary Figs. S1 and S2).

## Discussion

Cortical responses to near threshold letters differ from those elicited by Vernier offsets in their response topography and temporal dynamics, indicating that they are generated by different underlying sources. Importantly, the earliest letter-related activity—measured as the RC1 1F response—is maximal over left lateral occipital electrodes while the later-in-time RC2 letter 1F response is distributed more posteriorly. Source analysis suggests that the structure of letters is first extracted in a higher-order visual area, such as the VWFA or IOG^44,45^, and that the later activity seen on posterior electrodes may be the result of feedback. Prior studies using Visual Evoked Potentials/Fields have also found left-lateralized responses to letter/word forms compared to matched non-letter stimuli^45,46,53–60^, but have not identified the later occipital component we find here. By contrast, the dominant component of the Vernier-specific 1F response is maximal focally over Oz and its more broadly distributed RC2 occurs later, consistent with a feedforward progression from early visual cortex to higher-order areas.

The fMRI literature suggests that letters are predominantly processed by ventral occipital temporal cortex^39–43,61^, with a left hemisphere dominance. Weaker fMRI activations have also been reported in the IOG that are also left-lateralized^62–64^. Intra-cranial EEG and extra-cranially recorded MEG responses to letter *vs* pseudo-fonts have also been localized to the IOG as well as more ventral sources consistent with the VWFA^44,45^. Our simulations with VWFA and IOG sources show that these ROIs each generate scalp fields that aresimilar to those we observe for RC1 at 1F (see supplementary Fig. S3A-B). Source analysis indicates that both ROIs have monotonically increasing, left-hemisphere-biased 1F responses (Supplementary Fig. S3D). However, an analysis of cross-talk between these two ROIs indicates that activity generated solely in one of these two areas can also appear in the other (Supplementary Fig S3C). Thus, our scalp-recorded results cannot on their own distinguish activity generated by these two second-tier extra-striate areas.

FMRI studies have only exceptionally^65^ reported letter-related activity in early visual areas that could underlie the 1F letter response indexed by RC2. We confirmed the early occipital nature of this source by localizing the raw 1F response in a sub-group of participants using a Minimum Norm inverse solution (Fig. S3). Source analysis recapitulated the size tuning seen for RC2 in the V1 ROI. There may be several reasons that the majority of fMRI studies have not found letter-related activity in early visual areas as we have. First, our SSVEP paradigm differs substantially from the event-related designs typically used in fMRI studies. We used 3 Hz periodic alternations of intact and scrambled letters, while the event-related designs have typically presented intact and scrambled letters in blocks of one type at slower presentation rates. The temporally dense presentation we use may elicit non-linear activity that is not generated in the temporally sparser designs used for fMRI or our presentation mode may generate top-down predictive responses due to the stimulus periodicity. It is also possible that the SSVEP has higher sensitivity to activity in early visual areas that likely underlies the letter 1F response indexed by RC2. Finally, this response in early visual areas may be related to local features in our scrambled letters that are not fully controlled by the global nature of the scrambling algorithm. For instance, while the scrambling algorithm equates the amplitudes of spatial frequencies, the phase relationships between spatial frequencies are not preserved. This results in a loss of spatial regularity in the scrambled letters. Future fMRI studies could use periodic alternations between intact and scrambled exemplars to test the temporal non-linearity and predictive coding hypotheses with greater spatial precision than afforded by scalp EEG.

The topography of the two Vernier 1F components we measured is consistent with them being generated in early visual areas, as seen in previous single-unit studies in macaque that use related stimuli^32,33^ and a prior EEG source-imaging study^38^ that used nearly identical stimuli as in the present study. Partial support for a feed-forward Vernier response network comes from the latter study’s finding that activity in a V1 ROI led that measured in a V4 ROI. A previous study has compared Vernier-related source distributions, with the same stimulation we used here, to those for contrast modulated gratings^38^. The two stimuli evoked a similar medial-occipital scalp topography that contrasts with the left-lateralized topography we observe with letters.

### Possible limitations on timing measurements

The absolute phase of RC1 for letters is nearly constant as a function of letter size and is ~ 90 deg, which corresponds to a latency of ~ 80 ms (see Fig. 4A). However, SSVEPs are periodic paradigms and are subject to wrap-around effects. As a result, a 90 deg phase delay could also correspond to a modulo of 2pi or 360 *degrees* phase shift, which in our case of 3 Hz stimuli, results in a latency of ~ 440 ms. This would place RC1 after RC2 rather than before it. Nonetheless, a delay of this magnitude is unlikely, given the much shorter latencies observed in transient ERP studies of word/letter processing^56,58–60^, which all show effects well before 200 ms. Also, intra-cranial recordings from VWFA showed an onset latency of ~ 130 ms for differential response to false fonts vs consonants^45^.

Alternatively, SSVEP phase estimates are subject to potential 180 *degree* uncertainties due to source orientation inversion. Transient ERP studies typically find letter-related effects that manifest as a modulation of a negative component that peaks around 150–170 ms (see above). Our response is a positive component rather than negative. It is possible that the topography of RC1 is polarity flipped, possibly due to a difference in source orientation in our paradigm. If this is the case, a delay of 167 ms (180 degrees phase shift) should be added to our estimate of letter response latency. This would result in a latency estimate of ~ 250 ms, which is again later than previous estimates from transient ERPs and the more direct estimate from intracranial electrodes. However, simulations presented in the Supplementary Materials indicate that the phase in VWFA and IOG ROIs is retained at the scalp and is not polarity inverted (see Fig S3B).

Our earlier delay of RC1 compared to previous studies, could be due to the use of steady-state vs transient stimulation, the use of full-pages *vs* foveally-fixated short letter strings, and/or the fact that we did not have semantically meaningful information in our stimuli. Differential intra-cranial responses to words *vs* consonants have an onset latency of ~ 200 ms compared to an onset latency of ~ 130 ms for consonants *vs* false-fonts^45^. This ~ 70 ms additional processing incurred with intelligible words is similar to the latency difference between our non-word/scrambled-word responses and word/scrambled word responses (~ 140 ms) measured with a similar SSVEP protocol^46^.

#### The role of other potential cues

The presence of offsets in the Vernier display can generate illusory contours. Because evoked responses can be generated by illusory contours^66–69^, it is possible that the response we measure could be generated by these contours rather than the offsets per se. An experiment in which the offsets are present but are jittered in a way that they do not support illusory contour generation would be needed to separate these possibilities. This experiment would help determine the level of processing being addressed by our Vernier task.

Our letter task uses texture synthesis methods to generate control images that we contrast with intact letter images. One could thus argue that we are studying texture rather than text-related responses. Texture-related activations have been measured in several fMRI studies^70–73^ but these studies have not shown the left-lateralization we observe for the 1F component, while text-related activations, by contrast do^39–43,73^.

### What links psychophysical Vernier and letter acuity?

An association between Vernier acuity and crowded letter acuity was first noted in the amblyopia literature where the two acuities were found to have nearly proportionate losses despite differing strongly in absolute value^11,20^. Subsequent studies replicated this empirical relationship^18,19^. The question thus arises as to what might link them. Vernier acuity is believed to be limited by cortical mechanisms because Vernier acuity can be rendered constant across the visual field if the stimuli are scaled by estimates of the cortical, rather than retinal magnification factor^23^. The loss of letter acuity that is the hallmark of amblyopia is the result of damage to cortical mechanisms^28–31^, so the two share a dependence on cortical processing, but this is not a strong mechanistic constraint in and of itself.

One factor that may link Vernier acuity and the amblyopic letter acuity deficit is a common dependence on retinal eccentricity. Vernier acuity thresholds double at an eccentricity of ~ 0.7 deg, their so-called E2 value^23,74,75^. The E2 value for crowded optotypes and words also falls steeply, with typical values well under 1 ^25–27,76,77^. By contrast acuity for single optotypes^26,76–81^ has an E2 value of ~ 1–2 deg *e.g*. values shallower than that for Vernier acuity but steeper than for grating acuity, which has an E2 of ~ 2.5^23,81^. Spatial interference effects are also present for Vernier acuity, particularly in the periphery^23,82^. Letter acuity measured with the array format is likely to be subject to spatial interference effects that are present during psychophysical acuity estimation under crowded conditions. Our arrays used a one letter-width inter-letter spacing at all letter sizes, resulting in the same constant crowding conditions as in the commonly used Bailey-Lovie LogMAR acuity chart^3^. This spacing results in strong spatial interference effects on letter acuity for non-foveolar letters^26^.

The steep eccentricity dependence of Vernier acuity and crowded letter acuity make them susceptible to the pathological processes in amblyopia that depress functioning in the central visual field. These pathological processes include loss of high-spatial frequency (foveal) receptive fields^83–85^ and/or abnormal binocular inhibitory interactions (suppression), which are strongest in central vision^86–89^. The linkage of performance in the two tasks in amblyopia may thus be at least in part due to amblyogenic processes that are strongest in the central visual field rather than an explicit sharing of common neural mechanisms.

Our source analysis indicates that the earliest latency letter- and Vernier-related activities are recorded in distinct cortical areas. Our analysis, however, also suggests that the two functions may share a common substrate in early visual cortex that manifests in the RC2 1F response. Evidence from human fMRI suggests that the effects of amblyopia can be seen in early visual areas^90,91^. Amblyopia may act on the common RC2 substrate (or an even earlier one) and provide a mechanism through which the two responses could covary in amblyopia. It is important to note that while Vernier acuity and letter acuity are similarly affected across the broad spectrum of amblyopia sub-types, the slope of the regression line linking Vernier and letter acuities (by taking into account their variances) is slightly different in strabismic and anisometropic sub-groups. Vernier acuity in anisometropic amblyopia is worse than the expected value based on their letter acuity, while in strabismus, it is better than the expected value^18^. This suggests possible different underlying neural bases for strabismic and anisometropic amblyopia that could be explored with the neural measures we present here. Furthermore, given the different underlying sources, it would not be surprising to find cases where letter processing is disrupted independently of Vernier acuity. This pattern has been reported in dyslexia, for example^92^.

## Methods

### Participants

Eighteen participants enrolled in this study (Seven females and eleven males; age ranged from 20 to 67, four left-handers). All participants had normal or corrected to normal visual acuity as measured with a Bailey–Lovie constant LogMAR chart with five letters per line. All participants had stereo acuity better than 40 arcsec as measured with the RandDot stereotest (Stereo Optical Co., Inc., Chicago, IL). The study was approved by the Institutional Review Board of Stanford University and conformed to the tenets of the Declaration of Helsinki. All participants provided informed written consent prior to the study after the procedures were explained to them.

## Visual stimuli

The visual stimuli consisted of graded series of Vernier offsets and letter sizes presented within an SSVEP paradigm. The Vernier and letter stimuli each alternated between two states at a rate of 3 Hz as illustrated schematically in Fig. 1. The stimulus alternations were frame-precise and were synchronized with the data acquisition at sub-millisecond precision. The Vernier offset stimuli comprised a base pattern consisting of a 2 c/deg square-wave grating into which a set of Vernier offsets was periodically introduced and withdrawn. The displaced sections of the grating were 2 deg in height and alternated with static bands of the same height. The offset sizes were presented at five equal log steps in separate stimulus conditions. Offset values ranged between 1.4 and 5.6 arcmins (0.15 to 0.75 LogMAR). The Vernier stimuli extended 6° in each direction vertically and horizontally from a fixation point in the center of the screen. The stimuli were presented at 90% contrast at a mean luminance of 50 cd/m^2^.

The letter stimuli comprised a base pattern of nonsense letters of varying font size alternated with rows of intact random letter images of the same spatial scale. The intact letter images were rendered in the Sloan font (https://github.com/denispelli/Eye-Chart-Fonts/blob/master/Sloan.otf) with between-line and between-letter spacing of one letter width/height. Scrambled letter images were generated from the intact letter images by applying a texture synthesis algorithm^47^ that is available at https://www.cns.nyu.edu/~lcv/texture/. This algorithm synthesizes new textures from arrays of intact letters, which are also textures. The algorithm preserves low- and mid-level image statistics by first learning the joint distribution of filter locations, orientations, and scales from the intact letter arrays. These distributions are then used to adjust pixel values of random input images until they match the joint histograms of the intact letter arrays. A human fMRI study^93^ has reported that V1 and V2 do not respond differentially to intact vs scrambled natural images. Letter stimuli extended 4.5° in each direction vertically and 8° in each direction horizontally from a fixation point in the center of the screen, resulting in stimuli of 9° × 16° size. The logMAR acuity of letters was presented in five equal steps from 0.15 and 1.06 in separate stimulus conditions. The images used for letter array and scrambled letters varied randomly between each alternation.

### Experimental procedure

The participants were instructed to fixate the center of the screen and to refrain from blinking during individual trials that lasted 12 s. The experiment divided into 8 blocks, each of which included 2 twelve-second trials for each of the 10 conditions (5 Vernier and 5 letter conditions, *e.g.* twenty 12 s trials per block). These trials were presented with a random sequence within each block. In total, 16 trials were recorded for each condition.

### EEG data acquisition and processing

EEG data was acquired using EGI NetStation software and 128-channel EGI SensorNets (Electrical Geodesics Inc., Eugene, OR) at a sampling frequency of 500 Hz, with Cz as reference electrode. The EEG was then resampled to 420 Hz, to have integer number of data samples per stimulus cycle (140 samples per cycle) and exported and bandpass filtered between 0.3 and 50 Hz. Data preprocessing was then performed offline using an in-house software. Artifact rejection involved an electrode replacement procedure for noisy channels by averaging over six neighboring channels. The EEG data was then common average referenced, and epochs with significant number of data samples exceeding a threshold for artifact detection (30–80 μV) were excluded (on average 9% of trials were excluded).

### Spectral analysis

For analysis of the SSVEP data, the first second of each trial’s data record was excluded to allow for the visual system to attain the steady-state response condition. The data for the subsequent 10 s of each trial were divided into 2-s epochs and were submitted to a Discrete Fourier Transform that resulted in 0.5 Hz frequency resolution. The 2-s epochs contained an exact integer number of cycles (6) of the stimulus frequency and each cycle had an integer number of data samples, providing exact bin-centering of the Fourier transformed data. The complex-valued data were then averaged coherently in the frequency domain over the 5 epoch per trial and the 20 trials per block.

Harmonic analysis of the SSVEP was used to differentiate response components sensitive to configural aspects of the evoked response (*e.g.* the first harmonic (1F) component of the response that encodes differences between intact vs scrambled letters and aligned vs misaligned gratings) from the one that contains a mixture of activity that is not specifically configural, e.g. the second harmonic (2F) response to contrast change at image updates^52^.

### Reliable component analysis

To determine if there was more than one underlying source of 1F and 2F SSVEP activity, we decomposed the sensor data into a set of physiologically interpretable components using Reliable Components Analysis (RCA)^94^. RCA is a spatial filtering technique that decomposes the 128-channel montage into a small number of components by maximizing trial-to-trial consistency. This is done by solving a generalized eigenvalue problem on the cross-trial covariance matrices. Each RC component is characterized by a set of sensor weights that are used to transfer the data from sensor space into component space. RCA is an appropriate decomposition method because the SSVEP signal has constant response phase over repeated trials of the same stimulus. Additive experimental noise from the background EEG and other extraneous sources is thus de-emphasized by weighting the electrodes to maximize trial-to-trial consistency.

The real and imaginary values of Fourier coefficients calculated over each 2-s epochs, across the 128 sensors, and across trials and participants, served as the input data for the two RCA analysis. Here, we applied RCA separately on Fourier coefficients from first (3 Hz) and second (6 Hz) harmonics of stimulus frequency, to distinguish the configural and non-configural sources underlying Vernier and letter acuities.

### Statistical analysis

In order to determine whether 1F and 2F responses in the RC1 and RC2 components were statistically reliable, we compared the amplitudes at 1F and 2F to the experimentally determined noise level. We calculated the ratio of signal power at 1F or 2F frequencies (3 or 6 Hz) to the average power at two neighboring frequency bins (2.5 and 3.5 Hz for 1F and 5.5 and 6.5 Hz for 2F) that had no evoked power. The SNR values are reported in dB which is 10log10 of the power SNR values. We calculated a paired t-test to assess the statistical significance of the power SNR estimates. Here, signal and noise amplitudes were averaged over 5 logMARs and *t* test was run for each of RC1 and RC2 of Vernier and letter targets separately. Note that this is a conservative test of the presence of a signal, as it is not a test against zero signal, rather a test against the additive EEG noise floor.

To compare the amplitude and phase tunings of RC1 and RC2 for each of the two acuity targets, we used a two-way repeated measure ANOVA, fitted with a linear mixed-model, with reliable component as the first within-group factor (with RC1 and RC2 levels), and logMAR as the second within-group factor (with 5 levels equal to 5 logMAR sizes) and amplitude or phase of the RCs as the dependent variable.

## Supplementary information

Supplementary Information.

## Data Availability

The dataset analyzed in the current study is available upon request from the corresponding author.
