# Supplementary material for: Neural sources of letter and Vernier acuity
Source: Sci Rep. 2020 Sep 22;10:15449. doi: 10.1038/s41598-020-72370-3 (PMC7509830; doi:10.1038/s41598-020-72370-3)
Supplement: Supplementary file 1 — Supplementary Information. [file 41598_2020_72370_MOESM1_ESM.pdf]

Neural sources of Letter and Vernier acuity

Elham Barzegaran

and

Anthony M. Norcia

Wu Tsai Neurosciences Institute, Stanford University

290 Jane Stanford Way, Stanford, CA 94305

***Corresponding Authors:** Anthony M. Norcia, amnorcia@stanford.edu

Elham Barzegaran, e.barzegaran@gmail.com

Supplementary materials

1. Reliable Component Analysis of 2F responses

Responses to local contrast changes manifest at 2F and the 2F response patterns across stimulus conditions are strikingly different from those for 1F for both Letter and Vernier tasks as can be seen by comparing Fig. 4 of the main paper to Fig. S1 here. For the letter targets, the earliest response manifested in RC2 which is focally distributed over the occipital pole (electrode 75) while at 1F RC1 is the earlier component and it is maximal on lateral occipital electrodes. The amplitude of the 2F RC2 response increases as a function of letter size up to a maximum at logMAR 0.84 rather than 0.6 for the RC2 response at 1F. The later, more broadly distributed 2F RC1 component is located over lateral occipital electrodes, with a maximum over right hemisphere (electrode 90), This pattern is consistent with a feed-forward pathway for 2F instead of a feedback pathway for 1F. The amplitude of RC1 does not change as a function of letter size, but it is monotonically increasing for 1F, further indicating that 1F and 2F are generated by different sources.

The Vernier RC2 at 2F is also distributed on the occipital midline, more anteriorly compared to that for letter targets, with maximum over electrodes 71 and 76 (Fig.S1). For the Vernier responses, RC2 leads RC1, which has distinct bilateral maxima at lateral occipital leads, with maxima over right electrodes (electrodes 90 and 91), consistent with a feedforward pathway. The amplitude of both RC1 and RC2 increased as a function of offset size, however, RC1 has a steeper slope.

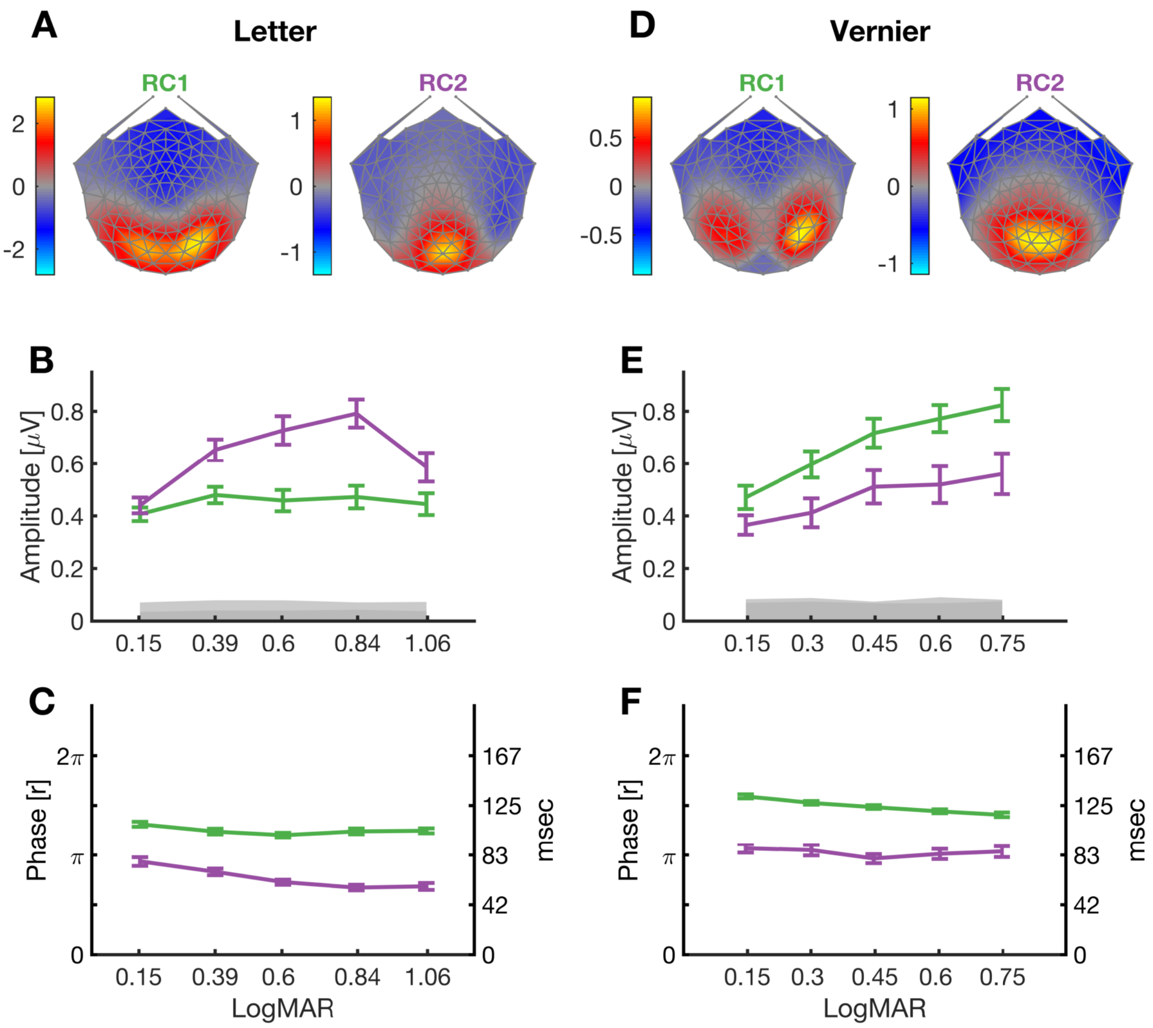

Figure S1. RCA weights, amplitudes and phases of 2F response component for letter and Vernier targets. ***A.*** *Letter task 2F RC1 topography (left) is maximal over right lateral occipital electrodes, RC2 topography (right) is more posteriorly distributed over the occipital pole.* ***B.*** *Letter task 2F amplitude as a function of increasing font size for RC1 (green) and RC2 (purple).* ***C.*** *Letter task 2F phase as a function of increasing font size for RC1 (green) and RC2 (purple).* ***D.*** *Vernier task 2F RC1 topography (left) is distributed over lateral occipital electrodes with right hemisphere bias. RC2 topography (right) is distributed over the parieto-occipital areas.* ***E.*** *Vernier task 2F amplitude as a function of increasing offset size for RC1 (green) and RC2 (purple).* ***F.*** *Vernier task 2F phase as a function of increasing offset size for RC1 (green) and RC2 (purple). The color bars in A and D indicate the weightings of RC1 and RC2 topographies. The mean noise level measured as the mean amplitude of the adjacent frequency bins to 2F, e.g. the mean amplitude of 5.5 and 6.5 H) is indicated by the grey area.*

Dynamics of the RC components of 2F. For letter targets, the response of RC2 leads the RC1 response by ~ 30 msec at the smallest letter size and by ~45 msec at the largest letter size (Fig. S2). For the Vernier targets, RC2 leads the RC1 response by ~45 msec for the smallest offset and by ~30 msec for the largest offset. The results further corroborate the feedforward nature of the 2F response.

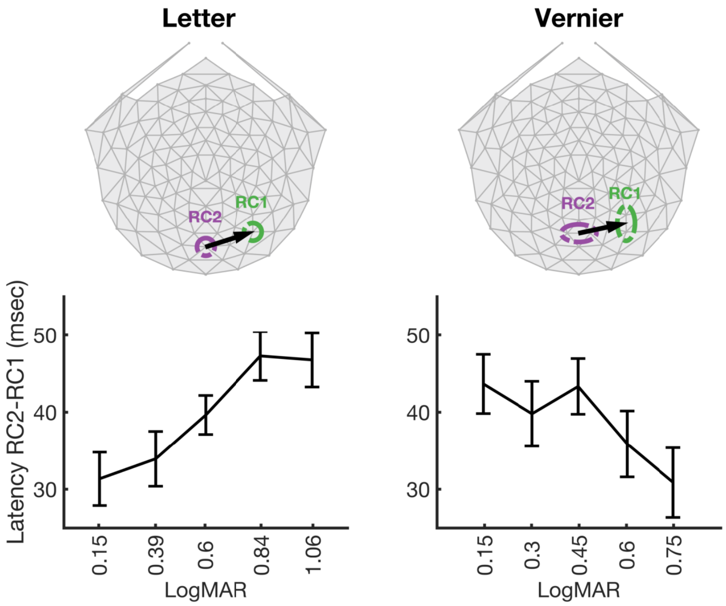

Figure S2. Response latency differences of RC1 and RC2 of 2F harmonic. Left. In letter target, RC2 with maximum amplitude over electrode 75 (purple dashed ellipse), leads RC1 with maximum over electrode 90 (green dashed ellipse). The latency difference between RC2 and RC1 is presented in msec as a function of letter size (in logMAR) in lower row. **Right.** In Vernier target, RC2 with maxima over electrodes 71 and 76 (purple dashed ellipse) leads RC1 with maxima over electrodes 90 and 91 (green dashed ellipse).

2. Cortical Sources of letter 1F responses

EEG forward and inverse modeling. Source analysis was conducted on a subset (n=10l 4 female) of the 18 participants in our experiment who had structural MRI data. The MRI data was collected using a General Electric Discovery 750 (General Electric Healthcare) equipped with a 32-channel head coil (Nova Medical) at the Center for Cognitive and Neurobiological Imaging at Stanford University. The MRI data acquisition included T1-weighted images (1.0 × 1.0 × 1.0 mm resolution, TE = 2.5 ms, TR = 6.6 ms, flip angle = 12, FOV = 256 × 256) and one single whole-brain T2-weighted structural image (1.0 × 1.0 × 1.0 mm resolution, TE = 75 ms, TR = 2500 ms, flip angle = 90, FOV = 256 × 256). During the EEG session, we digitized the electrode locations using Polhemus FASTRACK system to register the electrical and anatomical data.

A mid-gray cortical surface mesh was extracted using Freesurfer. Brain/skull, skull/scalp and scalp/air boundaries were extracted using FSL (http://www.fmrib.ox.ac.uk/fsl/). These boundaries, the mid-gray surface, and the digitized electrode locations were used to calculate a Boundary Element Method (BEM) forward model via MNE-suite (http://www.nmr.mgh.harvard.edu/martinos/userInfo/data/sofMNE.php). Current dipoles were located on 20484 uniformly distributed sources over cortical surface, with their orientation orthogonal to the local cortical surface. The forward solution was generated as a 20484×128 matrix for each participant. The details of forward and inverse modeling can be found in [2].

The EEG inverse solutions were estimated using a Minimum L2-norm (MN) inverse with a functional-area source correlation constraint (FACE)[3], that has been shown to improve the accuracy of visual areas inverse solutions.

Region of Interest (ROI) definitions. IOG and VWFA ROIs were defined in each participant using an atlas of functionally-defined ventral stream visual areas[4], and control ROIs were defined for each participant by functionally-defined probabilistic atlas of topographically organized visual areas[5]. These ROIs, defined on standardized surfaces, were converted to each participant's native space using AFNI's SurfToSurf function and

were down-sampled to match the surface meshes (20484 sources) used for forward/inverse solutions.

2.1. Source estimation of letter 1F responses using MN + FACE

To estimate the sources of the letter 1F responses, we applied the MN+FACE inverse solution to the FFT coefficients of the 1F frequency bin, and averaged the FFT coefficients coherently over the source vertices within the following visual ROIs: VWFA and IOG, and early visual ROIs V1, V2, V3.

Source analysis recovered increasing current density as letter size increased, with larger responses in the left than right hemisphere in IOG and VWFA ROIs as was seen in the tuning of RC1. By contrast, Letter size tuning in the early visual ROIs V1 and V2 was similar to the tuning of RC2 (compare figure Fig. S3 to Fig. 4 in the text).

2.2. Validation of source estimations of letter responses using simulation

To further investigate whether RC1 scalp activity for intact letters *vs* scrambled letters at 1F was consistent with activation of IOG and/or VWFA, we simulated the scalp topography expected from the independent activation of IOG, VWFA and control ROIs, V1, V2 and V3 using EEGSourceSim[2] (ESSim.Simulate.ResolutionMatrix function). We used the BEM forward models from the 10 participants that were used in the source estimation of 1F letter responses for this simulation. Scalp activity was simulated by placing unit current dipoles, oriented perpendicular to the local cortical surface at each vertex of the individually defined ROIs. Surface topographies for group data were generated by averaging the individual participant forward projections.

Fig. S3A-B shows the mean V1,V2, V3, IOG and VWFA forward projections along with the raw scalp topography of RC1 and RC2 for the 1F response for letters, reproduced from Fig. 2. The topographies of IOG and VWFA forward projections are broadly similar to each other and to the measured scalp field.

The similarity of the forward projections of the VWFA and IOG ROIs suggest that uniquely localizing the activity of the RC1 to one specific ROI is difficult. To quantify this, we computed cross-talk matrix using the same function from EEGSourceSim for VWFA,

IOG and control ROIs. Briefly, the cross-talk matrix is computed by placing unit current dipoles at the vertices of the source ROI and then measuring current density in the target ROIs. Perfect localization would result in activity being restricted to the diagonal of the cross-talk matrix, and presence of non-diagonal elements indicates that EEG inverse solution cannot perfectly unmix the activity of the ROIs. The matrix (Fig S3c) shows strong off-diagonal elements for IOG/VWFA ROIs in the left hemisphere (Cross-talk = 0.31). This is mainly due to the ill-posed nature of the EEG inverse solution and the fact that the VWFA and IOG are located in ventral surface of the brain, which are distant from EEG electrodes, therefore their localization error is higher.

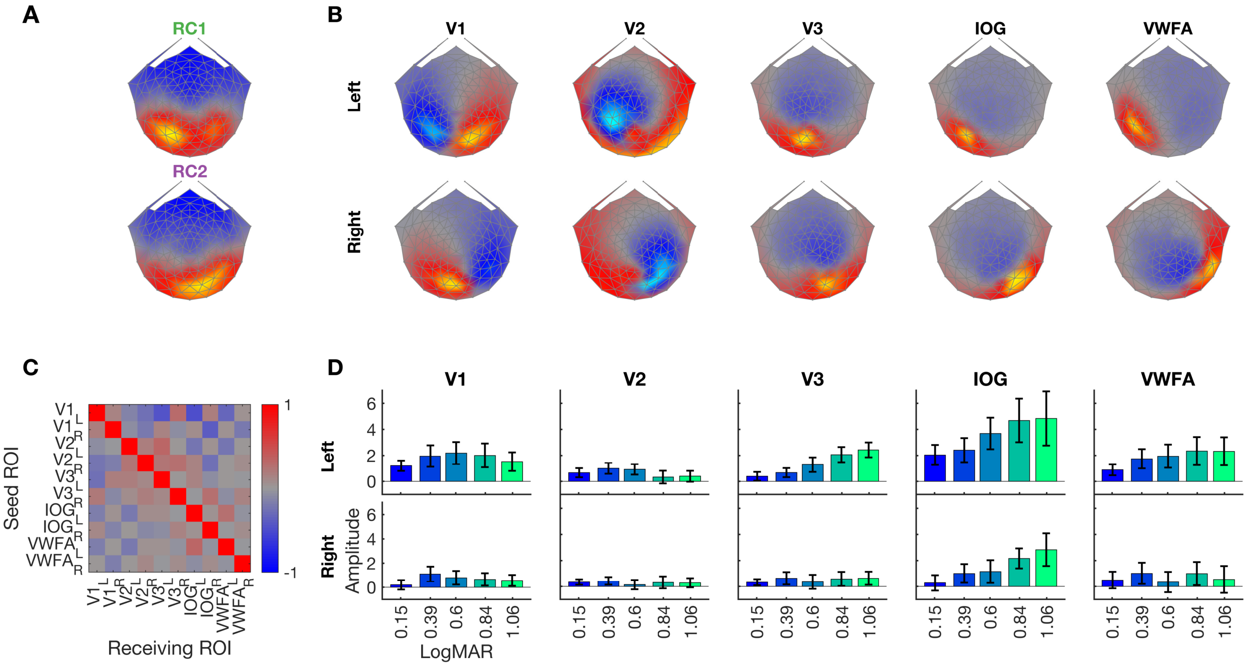

Figure S3. Source estimation of letter 1F responses. A. *Letter task 1F RCA topographies.* ***B.*** *Forward projections of five ROIs (Left and right hemispheres) using simulation. The maximum amplitude for left and right ROIs is located over electrodes 83 and 75 for V1, 88 and 68 for V2, 70 and 83 for V3, 65 and 90 for IOG, and 58 and 100 for VWFA.* ***C.*** *Cross-talk matrix for the selected ROIs, generated by simulation. The color scale is presented on the right.* ***D.*** *Average current source densities for the five ROIs, calculated by application of MN+FACE inverse solution to 1F letter task responses.*

References

1 Stuart, J. A. & Burian, H. M. A study of separation difficulty. Its relationship to visual acuity in normal and amblyopic eyes. *Am J Ophthalmol* **53**, 471-477 (1962).

2 Barzegaran, E., Bosse, S., Kohler, P. J. & Norcia, A. M. EEGSourceSim: A framework for realistic simulation of EEG scalp data using MRI-based forward models and biologically plausible signals and noise. *Journal of Neuroscience Methods* **328**, 108377, doi:https://doi.org/10.1016/j.jneumeth.2019.108377 (2019).

3 Cottereau, B. R., Ales, J. M. & Norcia, A. M. Increasing the accuracy of electromagnetic inverses using functional area source correlation constraints. *Human brain mapping* **33**, 2694-2713 (2012).

4 Weiner, K. S. *et al.* The cytoarchitecture of domain-specific regions in human high-level visual cortex. *Cerebral cortex* **27**, 146-161 (2017).

5 Wang, L., Mruczek, R. E., Arcaro, M. J. & Kastner, S. Probabilistic maps of visual topography in human cortex. *Cerebral cortex* **25**, 3911-3931 (2014).
